# Emergence of unusual species of enterococci causing infections, South India

**DOI:** 10.1186/1471-2334-5-14

**Published:** 2005-03-17

**Authors:** Vittal P Prakash, Sambasiva R Rao, Subash C Parija

**Affiliations:** 1 Department of Microbiology, Jawaharlal Institute of Postgraduate Medical Education and Research (JIPMER), Pondicherry, India; 2 Vice-Chancellor, NTR University of Health Sciences, Vijayawada, India

## Abstract

**Background:**

Enterococci tend to be one of the leading causes of nosocomial infections, with *E. faecalis *and *E. faecium *accounting up to 90% of the clinical isolates. Nevertheless, the incidence of other species of enterococci from clinical sources shows an alarming increase with the properties of intrinsic resistance to several antibiotics including beta-lactams and glycopeptides. Thus proper identification of enterococci to species level is quintessential for management and prevention of these bacteria in any healthcare facility. Hence this work was undertaken to study the prevalence of unusual species of enterococci causing human infections, in a tertiary care hospital in South India.

**Methods:**

The study was conducted in a tertiary care hospital in South India from July 2001 to June 2003. Isolates of enterococci were collected from various clinical specimens and speciated using extensive phenotypic and physiological tests. Antimicrobial susceptibility testing were performed and interpreted as per NCCLS guidelines. Whole cell protein (WCP) fingerprinting of enterococci were done for species validation by sodium dodecyl sulfate-polyacrylamide gel electrophoresis (SDS-PAGE) and analyzed computationally.

**Results:**

Our study showed the prevalence of unusual (non-faecalis and non-faecium enterococci) and atypical (biochemical variant) species of enterococci as 19% (46 isolates) and 5% (12 isolates) respectively. The 7 unusual species (46 isolates) isolated and confirmed by phenotypic characterization includes: 15 *E. gallinarum *(6.2%), 10 *E. avium *(4.1%), 6 *E. raffinosus *(2.5%), 6 *E. hirae *(2.5%), 4 *E. mundtii *(1.7%), 3 *E. casseliflavus*-including the two atypical isolates (1.2%) and 2 *E. durans *(0.8%). The 12 atypical enterococcal species (5%) that showed aberrant sugar reactions in conventional phenotyping were confirmed as *E. faecalis, E. faecium *and *E. casseliflavus *respectively by WCP fingerprinting. The antimicrobial susceptibility testing depicted the emergence of high-level aminoglycoside and beta-lactam resistance among different species apart from intrinsic vancomycin resistance by some species, while all the species tested were susceptible for linezolid and teicoplanin.

**Conclusion:**

Our study reveals the emergence of multi-drug resistance among unusual species of enterococci posing a serious therapeutic challenge. Precise identification of enterococci to species level enables us to access the species-specific antimicrobial resistance characteristics, apart from knowing the epidemiological pattern and their clinical significance in human infections.

## Background

Enterococci, generally regarded as normal flora of gastrointestinal and genitourinary tract of humans, have emerged as the etiogen of several nosocomial as well community-acquired infections since last two decades. Globally, many studies have revealed that enterococci tend to be one of the leading causes of several nosocomial infections, with the emergence and spread of multi drug resistance among isolates [1-3]. Since the inception of separate genus Enterococcus, there are 23 species of enterococci with clinical significance to date [4], of which *Enterococcus faecalis *and *Enterococcus faecium *accounts up to 90% of clinical isolates belonging to this genus [1]. Nevertheless, the incidence of other species of enterococci from clinical sources shows an alarming increase with the properties of intrinsic resistance to several antibiotics including beta-lactams and glycopeptides [5,6]. But the incidence of non-faecalis and non-faecium enterococci is underestimated because of frequent misidentification. On several instances only one phenotypic character differentiates one species from another, and to further complicate some strains of enterococci do not posses the exact phenotypic character of the type strains, and there comes confusion over their exact taxonomic status [7]. Thus proper identification of enterococci to species level is quintessential for management and prevention of these bacteria in any health care facility. Many studies focus on the two most common species *E. faecalis *and *E. faecium*, and only few reports or studies of non-faecalis and non-faecium enterococci are prevalent [5,6]. Hence the aim of our study was to check the prevalence of unusual and atypical species of enterococci causing human infections, in a tertiary care hospital in South India over a time period.

## Methods

### i. Bacterial isolates and conventional phenotypic characterization of enterococci

The study was conducted in a 900-bedded tertiary care hospital at Pondicherry, South India from July 2001 to June 2003. Isolates of enterococci were collected over the time period from various clinical specimens such as blood, urine, wound swabs and pus (surgical and non-surgical), catheters, ascitic fluid, synovial fluid, by plating them on 5% Sheep Blood agar and Mac-conkey agar, as well on Bile esculin azide agar (Hi-media, Mumbai, India) as per nature of the specimen. Extensive phenotypic and physiological characterization was carried out by the conventional tests devised by Facklam et al [3,8]. Carbohydrate fermentation tests were performed using 1% sugar discs in Brain heart infusion (BHI) broth with Andrade's indicator (Hi-media, Mumbai, India) as per manufacturer's instructions. The following sugars were tested for fermentation by isolates using commercial discs: mannitol, sorbitol, inulin, arabinose, melibiose, sucrose, raffinose, trehalose, lactose, glycerol, salicin, maltose, adonitol, and xylose, while sorbose and ribose were added to a final concentration of 1% to the broth base directly after sterilization (due to non-availability of discs). Group D antigen was detected using a commercial latex agglutination kit (The Binding site limited, Birmingham, B29 6AT) as per manufacturer's™ instructions.

### ii. Antimicrobial susceptibility testing

Antibiotic susceptibility testing of the clinical isolates along with the quality control strains were performed using BHI agar instead of Muller Hinton agar by disk diffusion method (for the antibiotics: penicillin [10 units], ampicillin [10 μg], gentamicin-high content [120 μg], streptomycin-high content [300 μg], ciprofloxacin [5 μg], nitrofurantoin-for urinary isolates only [300 μg], vancomycin [30 μg], teicoplanin [30 μg] and linezolid [30 μg]), standard agar dilution (for the antibiotics mentioned in Table-1) and agar screening methods (for vancomycin and high-level aminoglycoside resistance) and interpreted as per NCCLS guidelines [9]. Production of β-lactamase was determined by using nitrocefin discs (BBL Microsystems) as per manufacturer's™ instructions.

**Table 1:** Analysis of MIC ranges of unusual species of enterococci.

Species tested (no.of.isolates)	Antibiotic Tested	No. of isolates at specified MIC, in μg/mL						Susc^a^,%
			
		2	4	8	16	32	≥ 64	
*E. avium *(10)	Pen.	9	9	9	9	7	6	10
	Amp.	9	6	6	6	6	0	40
	Van.	4	2	0	0	0	0	100
	Te.	0	0	0	0	0	0	100
	Va.Scr.	NA	NA	NA	NA	NA	NA	100
	HLGm.	NA	NA	NA	NA	NA	NA	10
	HLStr.	NA	NA	NA	NA	NA	NA	50

*E. casseliflavus *(3)	Pen.	0	0	0	0	0	0	100
	Amp.	0	0	0	0	0	0	100
	Van.	3	3	0	0	0	0	NA
	Te.	0	0	0	0	0	0	100
	Va.Scr.	NA	NA	NA	NA	NA	NA	66.6
	HLGm.	NA	NA	NA	NA	NA	NA	100
	HLStr.	NA	NA	NA	NA	NA	NA	100

*E. durans *(2)	Pen.	2	1	1	1	0	0	50
	Amp.	1	1	1	1	0	0	50
	Van.	2	1	0	0	0	0	100
	Te.	1	0	0	0	0	0	100
	Va.Scr.	NA	NA	NA	NA	NA	NA	50
	HLGm.	NA	NA	NA	NA	NA	NA	50
	HLStr.	NA	NA	NA	NA	NA	NA	0

*E. gallinarum *(15)	Pen.	9	9	8	8	8	8	46.6
	Amp.	8	8	8	8	7	6	46.6
	Van.	13	9	2	0	0	0	NA
	Te.	0	0	0	0	0	0	100
	Va.Scr.	NA	NA	NA	NA	NA	NA	53.3
	HLGm.	NA	NA	NA	NA	NA	NA	46.6
	HLStr.	NA	NA	NA	NA	NA	NA	66.6

*E. hirae *(6)	Pen.	3	3	2	2	0	0	66.6
	Amp.	6	2	2	2	0	0	66.6
	Van.	0	0	0	0	0	0	100
	Te.	0	0	0	0	0	0	100
	Va.Scr.	NA	NA	NA	NA	NA	NA	100
	HLGm.	NA	NA	NA	NA	NA	NA	100
	HLStr.	NA	NA	NA	NA	NA	NA	100

*E. mundtii *(4)	Pen.	1	1	1	0	0	0	100
	Amp.	0	0	0	0	0	0	100
	Van.	2	2	0	0	0	0	100
	Te.	0	0	0	0	0	0	100
	Va.Scr.	NA	NA	NA	NA	NA	NA	50
	HLGm.	NA	NA	NA	NA	NA	NA	100
	HLStr.	NA	NA	NA	NA	NA	NA	50

*E. raffinosus *(6)	Pen.	4	4	4	4	4	4	33.3
	Amp.	4	4	4	4	4	0	33.3
	Van.	0	0	0	0	0	0	100
	Te.	0	0	0	0	0	0	100
	Va.Scr.	NA	NA	NA	NA	NA	NA	100
	HLGm.	NA	NA	NA	NA	NA	NA	66.6
	HLStr.	NA	NA	NA	NA	NA	NA	66.6

NOTE:

^a ^Interpretations based on NCCLS guidelines,

NA-not applicable; Susc- Susceptiblity,

Pen-Penicillin; Amp-Ampicillin; Van-Vancomycin; Te-Teicoplanin,

Va. Scr- Vancomycin resistance (6 μg/mL) agar screening,

HLGm- High-level gentamicin resistance (500 μg/mL) agar screening,

HLStr- High-level streptomycin resistance (2000 μg/mL) agar screening,

### iii. Molecular phenotyping of enterococci

Whole cell protein (WCP) analysis of the enterococcal isolates, including atypical biochemical variants of enterococci and the reference/type strains of enterococci (a kind gift from Dr. Richard.R.Facklam, CDC, Atlanta, GA. USA) were done using sodium dodecyl sulfate-polyacrylamide gel electrophoresis (SDS-PAGE) as described previously with minor modifications [10,11] for species identification, as well confirmation of species identities of atypical strains. Briefly, enterococcal test strains were grown for 18 hours at 37°C on Trypticase soy agar with 5% sheep blood. The samples were prepared by removing the bacterial growth from the surface of agar plate carefully with a sterile disposable loop, and suspended in 5 ml of sterile saline solution in order to obtain a turbidity equal to that of No.8 MacFarland density standard, centrifuged, and resuspended in 0.5 ml of an aqueous lysozyme solution (10 mg/ml). The suspensions were incubated in a water bath preset at 37°C for 2 hours. The WCP extracts were obtained by mixing one part of whole cell extract to one part of sample loading buffer, and boiled for 5 minutes and separated by SDS-PAGE along with a broad range molecular weight marker (New England Biolabs Inc.,) as per standard procedure [11]. The SDS-PAGE was performed using 5% stacking gel and 10% separating gel at a constant current of 20 mA using a mini-gel electrophoresis system (Bangalore Genei, India) and stained with Coomassie brilliant blue. Visual comparisons of the gels were made, and documentation done using a Gel doc system (Vilber loubert, France) for further analysis. The gel images were analyzed and dendrogram constructed using appropriate software (Bionumerics, version 2.5, Applied Maths, Kortrijik, Belgium) for validating the taxonomy of the enterococcal species studied.

## Results

### Conventional and Molecular phenotyping of enterococci

**Figure 1:**
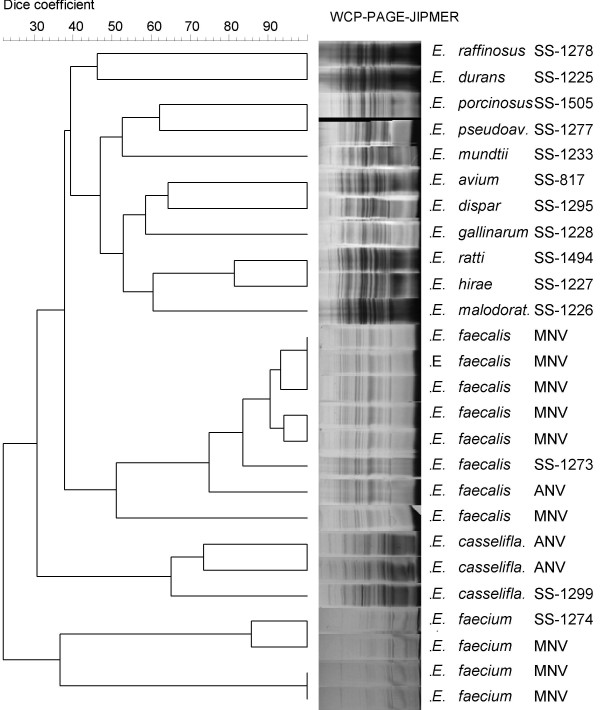
Cluster analysis of atypical strains of *Enterococci *using Dice coefficient and UPGMA method (Bionumerics, Applied Maths, Belgium). Note : SS- Designation of CDC standard strains, *E. porcinosus *is currently designated as *E. villorum*, *E. pseudoav.- E. pseudoavium*; *E. malodorat.- E. malodoratus*, *E. casselifla.- E. casseliflavus*, MNV- Mannitol negative variant; ANV- Arginine negative variant.

We isolated a total of 242 enterococci during our 2-year study period from different clinical samples. The biochemical phenotyping results revealed 46 isolates (19%) belonging to 7 different unusual species of enterococci (excluding *E. faecalis *and *E. faecium*-data not shown) which included 15 *E. gallinarum *(6.2%), 10 *E. avium *(4.1%), 6 *E. raffinosus *(2.5%), 6 *E. hirae *(2.5%), 4 *E. mundtii *(1.7%), 3 *E. casseliflavus *(1.2%) and 2 *E. durans *(0.8%). The distribution by site of isolation for the 46 unusual enterococcal species included 30 isolates- 12 *E. gallinarum*, 6 *E. avium*, 3 each of *E. hirae*, *E. casseliflavus *and *E. raffinosus*, 2 *E. mundtii *and 1 *E. durans *(65.2%) from bloodstream, 6 isolates- 3 *E. raffinosus*, 2 *E. avium *and 1 *E. mundtii *(13 %) from surgical and non-surgical wound swabs, 10 isolates- 3 each of *E. hirae *and *E. gallinarum*, 2 *E. avium *and 1 each of *E. durans *and *E. mundtii *(21.8%) from miscellaneous sites, including muscle tissues sent for anaerobic culture, catheter tips, peritoneal fluid, ear swab and urine. Of the 46 persons from whom unusual enterococci were obtained, 56.5% were males and 43.5% were females including newborn/neonates. The infections were polymicrobial in 6 (13%) of the 46 cases from which unusual enterococci were isolated, including 2 (6.7%) of 30 bloodstream infections. The 12 atypical enterococcal strains (5%) showing aberrant sugar reactions included 6 mannitol negative variant *E. faecalis *like species, 1 arginine negative variant *E. faecalis *like species, 3 mannitol negative variant *E. faecium *like species and, 2 arginine negative variant *E. casseliflavus *like species. The WCP analysis by SDS-PAGE confirmed the species identities of seven different species. Atypical strains showed a similar banding pattern like the reference strains from CDC (*E. faecalis *SS-1273, *E. faecium *SS-1274, *E. casseliflavus *SS-1229) except for minor quantitative differences, with no qualitative difference. The computational analysis of the WCP gel images of atypical strains were performed by Dice coefficient, and the dendrogram constructed using unweighted pair group method using arithmetic averages (UPGMA) as shown in Figure-1, and validated their exact taxonomic status as *E. faecalis, E. faecium *and *E. casseliflavus *respectively. The 2 (atypical) isolates of arginine negative variant *E. casseliflavus *like species after taxonomic validation were included as an unusual species of enterococci accounting to 3 *E. casseliflavus *isolated overall.

### Antimicrobial susceptibility testing

The antimicrobial susceptibilities of the isolates given in Table-1 depict the ranges of MICs for various antimicrobial agents tested by standard agar dilution, and agar screening methods. The *E. gallinarum *and *E. casseliflavus *isolates showed reduced susceptibility to lesser concentrations of vancomycin ranging 2–8 μg/ml. Other species were highly susceptible for vancomycin and teicoplanin except one isolate of *E. durans*. High-level aminoglycoside resistance for gentamicin and streptomycin was found absent only in *E. casseliflavus *and *E. hirae*, while other species exhibited variable susceptibilities ranging 0 – 66.7% for either aminoglycoside tested. The disk diffusion testing showed 100% susceptibility for linezolid and teicoplanin by all isolates tested, while *E. casseliflavus *and *E. mundtii *showed 100% susceptibility for penicillin and ampicillin. Only 37% of unusual enterococcal isolates were susceptible to ciprofloxacin, with resistance exhibited by 9 *E. avium *(n = 10), 2 *E. durans *(n = 2), 11 *E. gallinarum *(n = 15), 2 *E. hirae *(n = 6), 2 *E. mundtii *(n = 4) and, 3 *E. raffinosus *(n = 6). Only *E. casseliflavus *(n = 3) exhibited 100% susceptibility to ciprofloxacin. None of the 46 isolates was positive for β-lactamase, but resistance for β-lactam agents were prevalent variably among different species. The results of MICs for penicillin, ampicillin, high-level gentamicin and high-level streptomycin resistance were in accordance with the disk diffusion testing results except for vancomycin. Disk diffusion testing showed vancomycin resistance for 6 isolates (1 *E. durans*, 2 *E. mundtii*, 3 *E. gallinarum*), but the agar screening method exhibited vancomycin resistance for 11 isolates (2 *E. mundtii*, 1 *E. casseliflavus*, 1 *E. durans*, 7 *E. gallinarum*)(including 5 isolates-4 *E. gallinarum *and 1 *E. casseliflavus*, which showed susceptibility to vancomycin by the disk diffusion method).

## Discussion

Our study reveals that the prevalence of unusual species of enterococci as 19% in our clinical setup in South India. Many studies and reviews show the prevalence of non-faecalis and non-faecium enterococci as 2–10% [3,6,12]. Previous studies from India have reported *E. faecalis *and *E. faecium *as the only prevalent species [13-16], which may not reflect the true incidence rate. From our perspective the real incidence tends to be higher which in part can be explained as, misidentification of species due to exhibition of aberrant sugar reactions by some enterococci or, due to lack of application of the complete range of tests to identify non-faecalis and non-faecium enterococci [7,17]. The prevalence rate (19%) of our study was partly in accordance with another Indian study [18] showing 14.8% (excluding *E. faecalis *and *E. faecium*) prevalence of unusual species of enterococci from catheterized patients with urinary tract infections. *E. mundtii *and *E. durans *were not reported in their study, whose prevalence was 1.7% and 0.8% respectively in our study. *E. gallinarum *(6.2%) and *E. avium *(4.1%) were the most commonly identified species, which markedly differs in isolation rate (0.3–1.2%) from other studies [6,19,20]. The incidence of infections caused by unusual enterococcal species is of serious concern, since 43.5% of the isolates were from cases of septicemia without endocarditis. Apart from septicemia, the unusual species of enterococci were isolated frequently from cases of urinary tract infections, surgical and non-surgical wound infections and peritonitis. Most of the patients with the bloodstream infections had a peripheral or central catheter. Further, only 13% of enterococcal infections were polymicrobial, with majority from non-bloodstream isolates that underscores the clinical significance of these unusual enterococcal species. Although the unusual species of enterococci were isolated at regular intervals throughout our study period, we could find clustering of specific species during a specific time period from specific units/wards. Interestingly, 10 among the 15 isolates of *E. gallinarum *isolated during our study period were from pediatrics unit, while 7 of the 10 isolates exhibiting a similar antibiotype were isolated from the same ward within a span of 2 months. The remaining 3 of the 10 *E. gallinarum *were isolated from the same ward in the preceding 3 months, one of which showed an antibiotype similar to the cluster of 7 isolates. The same was the case of 3 *E. casseliflavus *isolated from the same pediatrics unit within a span of 2 months in the preceding year. Most of these (8 of 10 *E. gallinarum*, and all 3 *E. casseliflavus*) isolates were from cases of septicemia. Although molecular epidemiological studies have not been done to compare the genetic similarities of these isolates, the data depicts the nosocomial spread of these species. WCP analysis by SDS-PAGE had been proven to assist in validating the species identities as well, to identify strains that do not exhibit phenotypic characteristics identical to the type strains of each species [4,10,21]. We were able to validate the authenticity of the unusual species, and the exact taxonomic status of the atypical phenotypic variant strains identified by conventional biochemical testing as shown in Figure-1, using WCP fingerprinting by SDS-PAGE.

Ciprofloxacin resistance was 63% among isolates (excepting *E. casselifalvus*) which proves that it may be successful only in treating enterococcal urinary tract infections [1,9], since most of our isolates were from bloodstream and other related specimens. None of the isolates were β-lactamase producer, but penicillin and ampicillin resistance were exhibited by 54.3% and 45.7% isolates. We suggest penicillin binding protein modification based resistance for our isolates as a basis for β-lactam resistance, as depicted previously [22,23], but markedly differs from other Indian studies [15,24] showing up to 50% β-lactamase associated resistance. The prevalence of high-level gentamicin resistance (43.4%) and high-level streptomycin (37%) among unusual enterococcal isolates from our study partially correlates with studies from Japan [25] and United States [12,26]. In our study, most strains with high-level gentamicin resistance lacked high-level streptomycin resistance, and vice versa, thus facilitating the combination therapy (cell wall inhibitor plus aminoglycoside) treatment options for serious enterococcal bloodstream infections [1,9]. The prevalence of vancomycin resistance was 24% by agar screening /agar dilution method and 13% by disk diffusion. The difference may be attributed to the intrinsic low level vancomycin resistance (van C genotype), exhibited by 4 *E. gallinarum *and, 1 *E. casseliflavus *isolates, which may go undetected by disk diffusion testing [27]. Of serious concern was the low-level vancomycin resistance exhibited by one *E. durans *and two *E. mundtii *(MIC ≤ 6 μg/ml). The genotypic basis of vancomycin resistance for these 3 isolates yet to be studied, will give us a definitive picture regarding its clinical significance, since studies have reported the prevalence of vancomycin resistance in these two species, and its transferable nature from *E. durans *to *E. faecium *[28-30].

## Conclusion

Precise identification of enterococci to species level enables us, to access the species-specific antimicrobial resistance characteristics, apart from knowing the epidemiological pattern and their clinical significance in human infections. The difficulty in detecting (intrinsic) low-level vancomycin resistance by disk diffusion testing [28] emphasizes the necessity for including agar screening methods as per NCCLS guidelines in routine susceptibility testing of all enterococci isolated from clinical specimens [9]. Further as shown in our study, the increase in the rate of prevalence of the unusual and atypical species and the emergence of multidrug resistance among them, highlights the significance of rapid and accurate identification of enterococci to the species level for initiating appropriate therapeutic regimen, and reemphasizes the importance of the implementation of appropriate infection control measures to limit the nosocomial spread of these unusual species in any nosocomial setting.

## Competing interests

The author(s) declare that they have no competing interests.

## Authors' contributions

PVP designed the study and carried out the experimental works and analysis, and drafted the manuscript. RSR supervised and participated in the design of the study and coordination, and helped to draft the manuscript. SCP participated in the coordination of the study and helped to draft the manuscript.

## Pre-publication history

The pre-publication history for this paper can be accessed here:



## References

[B1] Murray BE (1990). The life and times of the enterococcus. Clin Microbiol Rev.

[B2] Fridkin SK, Gaynes RP (1999). Antimicrobial resistance in intensive care units. Clin Chest Med.

[B3] Facklam RR, Sahm DF, Texeira LM, Murray PR, Baron EJ, Pfaller MA, Tenover FC, Yolken RH (1999). Enterococcus. Manual of clinical microbiology.

[B4] Tyrrell GJ, Turnbull L, Teixeira LM, Lefebvre J, Carvalho Mda G, Facklam RR, Lovgren M (2002). *Enterococcus gilvus *sp. nov. and *Enterococcus pallens *sp. nov. isolated from human clinical specimens. J Clin Microbiol.

[B5] Dutka-Malen S, Evers S, Courvalin P (1995). Detection of glycopeptide resistance genotypes and identification to the species level of clinically relevant enterococci by PCR. J Clin Microbiol.

[B6] Gordon S, Swenson JM, Hill BC, Pigott NE, Facklam RR, Cooksey RC, Thornsberry C, Jarvis WR, Tenover FC (1992). Antimicrobial susceptibility patterns of common and unusual species of enterococci causing infections in the United States. Enterococcal Study Group. J Clin Microbiol.

[B7] Teixeira LM, Facklam RR, Steigerwalt AG, Pigott NE, Merquior VL, Brenner DJ (1995). Correlation between phenotypic characteristics and DNA relatedness within *Enterococcus faecium *strains. J Clin Microbiol.

[B8] Facklam RR, Collins MD (1989). Identification of *Enterococcus *species isolated from human infections by a conventional test scheme. J Clin Microbiol.

[B9] National Committee for Clinical Laboratory Standards (NCCLS) (2000). Methods for dilution antimicrobial susceptibility tests for bacteria that grow aerobically. Approved standard M7-A5 Wayne (PA).

[B10] Merquior VLC, Peralta JM, Facklam RR, Teixeira LM (1994). Analysis of electrophoretic whole-cell protein profiles as a tool for characterization of *Enterococcus *species. Curr Microbiol.

[B11] Laemmli UK (1970). Cleavage of structural proteins during the assembly of the head of bacteriophage T4. Nature.

[B12] Jones RN, Sader HS, Erwin ME, Anderson SC (1995). Emerging multiply resistant enterococci among clinical isolates. I. Prevalence data from 97 medical center surveillance study in the United States. *Enterococcus *Study Group. Diagn Microbiol Infect Dis.

[B13] Nischal M, Macaden R (1996). Biochemical speciation and haemolytic activity in enterococci. Indian Journal of Medical Microbiology.

[B14] Gulati V, Aggarwal A, Khanna S, Narang VK (1997). Biochemical speciation of enterococci causing human infections. Indian J Med Sci.

[B15] Devi PS, Rao PS, Shivananda PG (2002). Characterization, antibiotic susceptibility pattern and detection of beta-lactamases in Enterococci. Indian J Pathol Microbiol.

[B16] Bhat KG, Paul C, Bhat MG (1997). High level aminoglycoside resistance in enterococci isolated from hospitalized patients. Indian J Med Res.

[B17] Carvalho MG, Teixeira LM, Facklam RR (1998). Use of tests for acidification of methyl-alpha-D-glucopyranoside and susceptibility to efrotomycin for differentiation of strains of *Enterococcus *and some related genera. J Clin Microbiol.

[B18] Desai PJ, Pandit D, Mathur M, Gogate A (2001). Prevalence, identification and distribution of various species of enterococci isolated from clinical specimens with special reference to urinary tract infection in catheterized patients. Indian Journal of Medical Microbiology.

[B19] Low DE, Keller N, Barth A, Jones RN (2001). Clinical prevalence, antimicrobial susceptibility, and geographic resistance patterns of enterococci: results from the SENTRY Antimicrobial Surveillance Program, 1997–1999. Clin Infect Dis.

[B20] Pfaller MA, Jones RN, Doern GV, Sader HS, Kugler KC, Beach ML (1999). Survey of blood stream infections attributable to gram-positive cocci: frequency of occurrence and antimicrobial susceptibility of isolates collected in 1997 in the United States, Canada, and Latin America from the SENTRY Antimicrobial Surveillance Program. SENTRY Participants Group. Diagn Microbiol Infect Dis.

[B21] Vittal Prakash P, Sambasiva Rao R, Parija SC Molecular phenotyping of atypical Enterococcus species (Abstract). Proceedings of the XXVII National conference of Indian Association of Medical Microbiologists 5–9th November 2003, Mumbai, India.

[B22] Williamson R, LeBouguenec C, Gutmann L, Horaud T (1985). One or two low affinity penicillin-binding proteins may be responsible for the range of susceptibility of *Enterococcus faecium *to benzylpenicillin. J Gen Microbiol.

[B23] Fontana R, Ligozzi M, Pittaluga F, Satta G (1996). Intrinsic penicillin resistance in enterococci. Microb Drug Resist.

[B24] Parvathi S, Appala Raju B (2000). Comparative evaluation of beta lactamase production in enterococci by acidometric method and clover leaf technique. Indian Journal of Medical Microbiology.

[B25] Chiew YF, Tosaka M, Yamane N (1993). Prevalence of enterococcal high-level aminoglycoside resistance in Japan. Comparative detection by three methods. Diagn Microbiol Infect Dis.

[B26] Sahm DF, Boonlayangoor S, Schulz JE (1991). Detection of high-level aminoglycoside resistance in enterococci other than *Enterococcus faecalis*. J Clin Microbiol.

[B27] Swenson JM, Hill BC, Thornsberry C (1989). Problems with the disk diffusion test for detection of vancomycin resistance in enterococci. J Clin Microbiol.

[B28] Green M, Barbadora K, Michaels M (1991). Recovery of vancomycin-resistant gram-positive cocci from pediatric liver transplant recipients. J Clin Microbiol.

[B29] Cercenado E, Unal S, Eliopoulos CT, Rubin LG, Isenberg HD, Moellering RC, Eliopoulos GM (1995). Characterization of vancomycin resistance in *Enterococcus durans*. J Antimicrob Chemother.

[B30] Jenney A, Franklin C, Liolios L, Spelman D (2000). *Enterococcus durans *vanB. J Antimicrob Chemother.

